# Why do patients with ‘primary care sensitive’ problems access ambulance services? A systematic mapping review of the literature

**DOI:** 10.1136/bmjopen-2015-007726

**Published:** 2015-05-19

**Authors:** Matthew J Booker, Ali R G Shaw, Sarah Purdy

**Affiliations:** Centre for Academic Primary Care, School of Social and Community Medicine University of Bristol, Bristol, UK

**Keywords:** Ambulances, Emergency Medical Services, Urgent Care, Primary Health Care

## Abstract

**Objective:**

Emergency ambulance use for problems that could be managed in primary care continues to rise owing to complex reasons that are poorly understood. The objective of this systematic review is to draw together published evidence across a variety of study methodologies and settings to gain a better understanding of why patients seek help from ambulance services for these problems.

**Design:**

Systematic searches were undertaken across the MEDLINE, EMBASE, PsychINFO, CINAHL, Health Management Information Consortium and Health Management Information Service publication databases. Google Scholar, Web of Science, OpenSigle, EThOS and DART databases were also systematically searched for reports, proceedings, book chapters and theses, along with hand-searching of grey literature sources. Studies were included if they reported on findings examining patient, carer, health professional or service management interactions with ambulance services for primary care problems. All study methodologies and perspectives were of interest. Data were extracted, quality assessed and systematically mapped according to key findings through generation of an iterative framework.

**Results:**

A total of 31 studies met inclusion criteria. Findings were summarised across 5 broad categories: factors associated with individual patients; actions of care-givers and bystanders; population-level factors; health infrastructure factors; challenges faced by health professionals. A number of subcategories were developed to explore these factors in more detail.

**Conclusions:**

This review reports important factors that may impact on ambulance use for primary care problems across a global setting, including demographic measures associated with deprivation, minority status and individual social circumstances. Categorising ambulance calls for primary care problems as ‘inappropriate’ is context dependant and may be unhelpful. Potential implications for triage and risk management strategies are discussed.

**Strengths and limitations of this study**

- This is the first mapping review specifically exploring ambulance use among patients with problems amenable to management in primary care.
- This study was conducted according to rigorous systematic methodology in accordance with a prospectively published review protocol.
- The review is highly inclusive, summarising over 30 years of research evidence. This includes a range of global study settings, including qualitative, quantitative and mixed methods research.
- The heterogeneity of study methodologies and contexts presents a challenge in drawing together related and contrasting findings.
- There is relatively little research evidence addressing the specific question, reflected in the small number of studies fitting the quite inclusive review criteria.

## Introduction

The ways in which ambulance services are used have evolved significantly over the past two decades. Initially conceived as a system to transport the critically injured and unwell to hospital for emergency care,^1^ the majority of patient journeys are now no longer for cases of life-threatening injury or illness.^2^ Internationally, ambulance systems vary in the services they provide within the local health infrastructure.^1^ Many services are staffed by highly trained clinicians who are able to deliver advanced critical care at the scene of an incident, or provide enhanced medical treatment in a community setting. Others still perform a primarily transport role with more basic clinical capability. Despite these differences, the types of problems ambulances are being called for is changing. In the UK, demand for ambulances is rising at nearly 7% per annum.^3^ Contacts for conditions that would be amenable to management in primary care represent a substantial proportion of the workload.^4^ The reasons behind this shift are multifactorial and poorly understood. Previous work has focussed on reducing so-called ‘inappropriate’ use of ambulances. However, there is evidence to suggest the definition of ‘inappropriate’ is complex and context dependent.^5^ If services are to provide sustainable, safe and relevant care, an appreciation of what underpins the use of ambulances for primary care problems is vital.^4^ This systematic literature search and mapping review seeks to draw together published evidence across a mixture of methods with an international perspective, to summarise what is currently known about why ambulance services are used for primary care sensitive problems. This understanding will inform future urgent care service design.

## Methods

We undertook a systematic mapping review of published journal articles and relevant grey literature, exploring the question “Why do patients with ‘primary care sensitive’ problems seek help from ambulance services?” A systematic map is a review methodology that aims to map out and categorise literature on a particular topic with a view to undertaking further more detailed work,^6^ and is increasingly used in health services research and policy development.^7^ ^8^ This methodology is particularly useful for summarising and organising a broad, heterogeneous evidence base to identify a focus for more specific investigation.^9^ Our approach was based on the principles refined by the Social Care Institute for Excellence.^8^

### Search strategy

Searches were conducted on the following databases, for articles published between January 1980 and June 2014: MEDLINE, EMBASE, PsychINFO, CINAHL, Health Management Information Consortium and Health Management and Information Service. A Google Scholar and Web of Science search were undertaken to identify reports or proceedings not indexed in the above. Book chapters and theses were searched via the OpenSigle, EThOS and DART databases. Search terms were developed iteratively by discussion among the research team and a medical subject librarian, seeking a balance between comprehensiveness and focus. The final search strategy was piloted against a list of sample papers known to the research team to ensure that key references were reliably identified. The full review protocol and search strategy was published prospectively in the PROSPERO register (registration reference CRD42014009108). Forwards and backwards citation searching was undertaken with the aid of the reference management software EndNote (V. X7.1), with duplicate suppression. An updated search was undertaken prior to finalising the analysis. The comprehensive search strategy was supplemented with focussed hand-searches through key journals, and by approaching colleagues in collaborating institutions for relevant unpublished reports and ‘grey literature’. A total of 1424 documents were identified in the initial searching process.

### Inclusion and exclusion criteria

The inclusion criteria were articles published in the English language between 1980 and June 2014, reporting the findings of the research examining patient, carer, healthcare professional or health service management interactions with ambulance services for ‘primary care sensitive’ health problems. This includes the perspectives and experiences of patients (or their care-givers) who access ambulance services directly, and of the health professionals and service managers they encounter. Studies that reported on any stage of an ambulance contact episode (including emergency telephone call or referral, ambulance attendance, ambulance journey, clinical treatment and conveyance outcome) were of interest and thus included. The ‘primary care-related’ nature of the contact could be defined prospectively or retrospectively and from any perspective, either by explicit reference to terms related to primary care or family medicine, or by reference to a comprehensive list of indicator presentations, developed and piloted in conjunction with a medical subject librarian. This included presentations where mental health or social care were the primary need, where the consensus among the research team (two of whom are primary care clinicians) was that these could feasibly be encountered in a primary care setting.

The Phenomena of Interest and Context model^10^ underpinned the categorisation and construction of search terms, and as such, the full range of study methodologies and potential interventions were of interest. These included studies reporting qualitative methods, quantitative and mixed method analysis of routinely collected service data, clinical trials, service evaluations, and reviews of any/all of the above, with an additional interpretative element (eg, the development of ‘third order’ constructs). Studies were excluded if they only reported on routine primary care without any involvement of ambulance services or resources, or if English language translations were unavailable. It had been intended to exclude studies that were not undertaken in a health service providing some form of primary care model of healthcare. However, no studies were found to fulfil these criteria and, therefore, none were excluded on this basis.

### Reference screening

**Figure 1:**
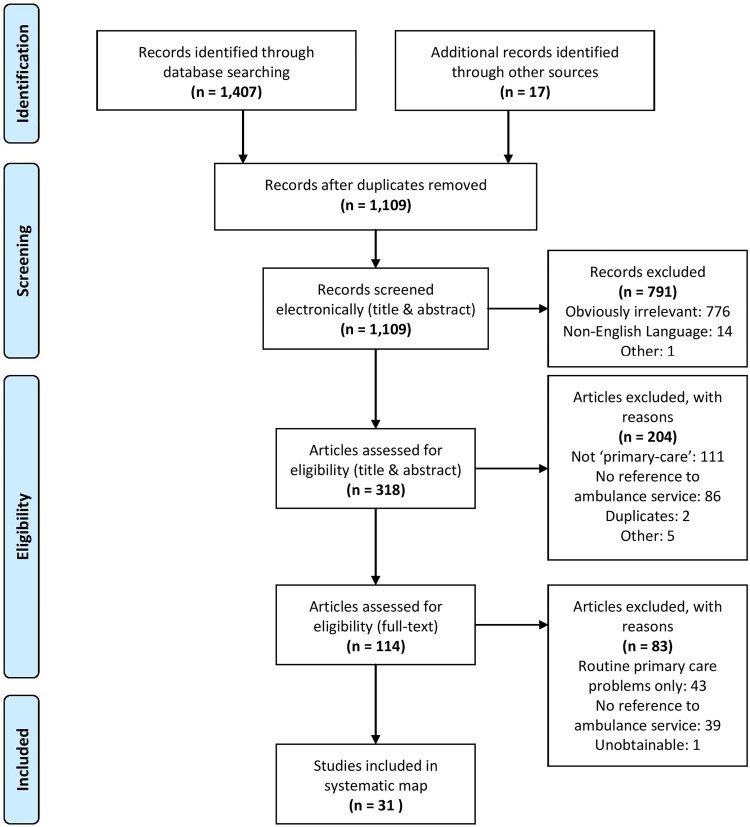
Preferred Reporting Items for Systematic Review and Meta-analysis (PRISMA) flow diagram for paper screening.

References and documents were managed with the aid of the reference management software EndNote (V. X7). A three-tier screening process was undertaken. The searches identified 1424 references. MJB initially suppressed duplicate references and removed incorrectly cited and foreign language references (n=14). The resulting list (n=1109) was then screened electronically, for obviously irrelevant papers, by title and abstract. The title and abstract of the remaining papers (n=318) were then screened against the eligibility criteria, and a sample (10%) of references excluded at this stage were independently verified by SP and AS with full agreement. The full papers (n=114) were then read and screened against the eligibility criteria independently by two researchers, with any disagreement resolved by consensus discussion with the third researcher. A total of 31 papers were included in the final systematic mapping process. The PRISMA flow diagram is shown in figure 1.

### Data extraction

Owing to the inclusive nature of this review, based on the relatively little relevant research literature, it was decided to include findings from studies of all methodologies. A customised data extraction tool was developed and piloted, based on a modification of the guidelines from the NHS Centre for Reviews and Dissemination.^11^ Standard author, date and citation data were extracted, along with details of setting, perspective and participants. Principal qualitative and quantitative findings were extracted, along with up to three ‘key messages’ from the discussion or conclusion sections (see online supplementary appendix table S1). One researcher (MJB) extracted the data, with verification undertaken by other members of the research team. Regular research meetings were held during the data extraction process, and any disagreement resolved by consensus discussion.

### Quality assessment

There are inherent complexities of evenly assessing ‘quality’ in a methodologically heterogeneous group of studies such as these,^12^ and debate in the literature about how to integrate the findings (if at all) of studies assessed as lower quality.^8^ ^13^ In this review, each study was assessed against the appropriate version of the Critical Appraisal Skills Programme checklist,^14^ which includes general and methodology-specific quality parameters. These tools were chosen as, with slight variations, they follow similar quality appraisal structures across a variety of quantitative study designs and qualitative research. This enables critical evaluation of quality according to a similar framework, despite the methodological heterogeneity. Following consensus discussion among the research team, none of the identified studies were excluded purely on quality grounds. However, limitations around study design and reporting were used to frame the discussion.

### Framework development

Following data extraction, an inductive mapping framework was developed in a process similar to established framework analysis as used in primary research.^15^ The findings and key messages were grouped categorically.

## Results

**Figure 2:**
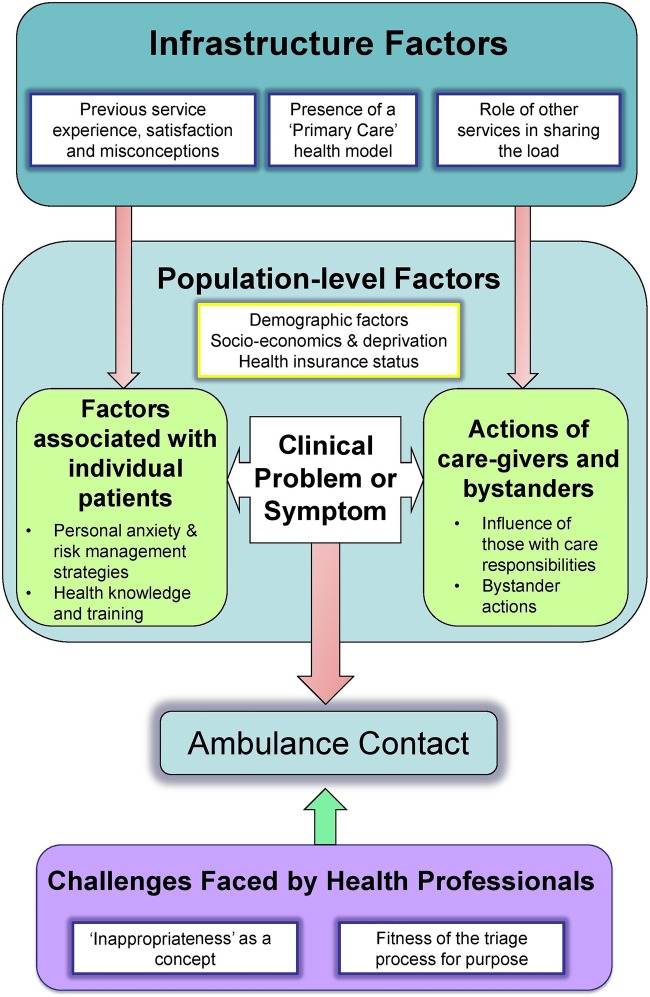
Relationships between categories derived from the mapping process.

We identified 31 papers relevant to this review. Table 1 summarises the characteristics of evidence included in the map. The framework resulted in five categories being identified, with 13 subcategories. Table 2 summarises the categories resulting from the mapping process, and table 3 the distribution of categories by principle study methodology. Figure 2 provides a diagrammatic representation of the inductive mapping framework, indicating how—in this analysis—key messages from the literature were principally centred around one of the three domains—the effects of population characteristics, healthcare infrastructure characteristics or the perceptions or actions of healthcare staff. The majority (n=26) of studies contributed key messages to several subcategories within each domain. Only a minority (n=5) provided key messages that spanned more than one of the three domains.

**Table 1:** Summary characteristics of included papers

Characteristic	Number of papers n (% of total)
Methodology	
Wholly qualitative	6 (19)
Wholly quantitative	9 (29)
Mixed methods	14 (45)
Comprehensive review	2 (6)
Study setting	
UK	12 (39)
USA	9 (29)
Japan	2 (6)
Sweden	2 (6)
Global	2 (6)
Canada	1 (3)
The Netherlands	1 (3)
Norway	1 (3)
Australia	1 (3)
Year of publication	
1980–1989	1 (3)
1990–1999	8 (26)
2000–2009	14 (45)
2009 onwards	8 (26)
Main perspective	
Patients and carers	10 (32)
Health professionals	21 (68)

**Table 2:** Categories and subcategories of evidence developed from the mapping process

Category (number of papers)	Subcategory (number of papers)
Factors associated with individual patients themselves (16)	Category of clinical problem or symptom (9) Personal anxiety and risk-management strategies (6) Health knowledge and training, including first aid skills (1)
Actions of care-givers and bystanders (5)	Influence of those with care responsibilities (4) Bystander actions (3)
Population-level factors (11)	Demographic factors (9) Socioeconomic factors and deprivation (5) Health insurance status (4)
Health infrastructure factors (10)	Experience, satisfaction and misconceptions of health infrastructure (5) Presence of a primary care health model (3) Role of other services in unmet needs, including social care (5)
Challenges faced by healthcare professionals (21)	‘Inappropriateness’ as a concept (17) Fitness of the triage process for purpose (4)

**Table 3:** Distribution of categories and subcategories according to paper methodology (⦿=1 study)

	Qualitative	Mixed methods	Quantitative
Factors associated with individual patients themselves			
Category of clinical problem or symptom		⦿⦿⦿⦿⦿	⦿⦿
Personal anxiety and risk-management strategies	⦿⦿⦿⦿⦿	⦿	
Health knowledge and training, including first aid skills		⦿	
Actions of care-givers and bystanders			
Influence of those with care responsibilities	⦿⦿⦿	⦿	
Bystander actions	⦿	⦿⦿	
Population-level factors			
Demographic factors	⦿⦿	⦿⦿⦿⦿⦿	⦿⦿
Socioeconomic factors and deprivation		⦿⦿⦿	⦿⦿
Health insurance status		⦿⦿⦿	⦿
Health infrastructure factors			
Experience, satisfaction and misconceptions of health infrastructure	⦿⦿⦿⦿	⦿	
Presence of primary care model		⦿⦿	⦿
Role of other services in unmet needs	⦿⦿	⦿⦿	⦿
Challenges faced by health professionals			
‘Inappropriateness’ as a concept	⦿⦿	⦿⦿⦿⦿⦿⦿⦿⦿	⦿⦿⦿⦿⦿
Fitness of the triage process for purpose	⦿⦿		⦿⦿

### Factors associated with individual patients (n=16 studies)

Notwithstanding the complexities of determining ‘appropriateness’ of ambulance use from a variety of care-giving and care-receiving standpoints, much of the literature explores how urgent and emergency clinical presentations are recognised and defined by the users of ambulance services, and how this shapes risk management strategies. Studies report wide variation in what both patients and carers deem to be clinical ‘emergencies’, and what would be appropriate self-management thresholds.

### Category of clinical problem

One cross-sectional US study^16^ suggests overall poor physical health status is associated with ambulance use for low-acuity conditions rather than specific symptoms or conditions. This is supported by a similar study showing increased inappropriate use with increasing number of comorbidities, regardless of the actual presenting symptom.^17^ However, another similarly designed study reports chest pain, respiratory conditions and cardiac symptoms as the most likely clinical problems associated with a true need for ambulance attendance,^18^ while a Swedish paper reports that nearly half of abdominal pain and urinary presentations to emergency services unnecessarily used ambulances.^19^

Psychiatric conditions, behavioural disorders and drug and alcohol misuse present particular challenges for ambulance services across the full range of global settings. US studies analysing routinely collected national data sets^20^ and undertaking case note reviews in emergency departments^21^ both report high levels of excess ambulance usage among these groups.

Despite these specific symptom-related or diagnosis-related associations, other papers show unclear relationships between the clinical symptom and need for ambulance transport. One comprehensive review of the appropriateness of ambulance transport concludes that there is relatively little variation across study methodologies of ambulance contacts determined as inappropriate on the basis of clinical diagnosis (30–55%).^5^ These authors highlight the pitfalls of making this assignment retrospectively *after* clinical assessment, suggesting that basing judgement of appropriateness on information available after the contact (ie, a firm diagnosis) has many limitations in seeking to explain variation by clinical symptoms. Authors of another comprehensive review^22^ suggest that medical classification of urgency based on physiological measures contrasts with patient classification based on psychosocial factors, rendering distinctions by illness type less informative.

In addition, there is no clear relationship in the literature between whether the ambulance request was deemed to be ‘appropriate’ or not and whether the treatment was for a traumatic injury or a medical symptom. A UK case note review and patient interview study suggests 50% of calls for trauma-related conditions were unnecessary, compared with 20% for medical conditions.^23^ A Swedish study using similar methodology assesses the figure for trauma to be just 17%.^19^

### Personal anxiety and risk management strategy

Two interview studies^24^ ^25^ with patients from the UK highlight the importance of an individual's risk management strategies when choosing ambulance care. One thematic analysis identifies patient and carer anxieties surrounding the relatively small risks associated with choosing alternative care options.^24^ Another identifies the process of recognising the need for help, overemphasising the urgency, and therefore accepting that only ambulance-based care will meet this need.^25^ Feeling isolated or alone during this decision-making process is the criterion in choosing the immediacy of an ambulance response in this analysis.^25^ Studies that included surveys of patients at the time of ambulance care report a genuine fear of a life-threatening condition in 60% of patients,^26^ but frequently acknowledge the role of lay bystanders in influencing this assessment.^27^

One UK study^28^ set in a rural area explored pre-emptive strategies used by patients to mitigate the perceived risks, suggesting that a common practice in this cohort was to arrange routine primary care appointments early in an illness just in case they became necessary. A different UK study^29^ that explored ambulance paramedic views through focus groups reports that some ambulance clinicians feel unsupported by their employers in leaving patients at home after a call, suggesting that appropriate risk management is a challenge for both providers and consumers of ambulance care.

### Health knowledge

Only one UK study^30^ attempted to formally account for patient medical knowledge or training via online hypothetical case-vignettes to explore decision-making. This found a negative association between medically unnecessary ambulance use and formal first-aid training, and suggested that national first-aid training programmes may help the population discriminate between routine and emergency problems.

### Actions of care-givers and bystanders (n=5 studies)

Of those studies that formally included the perspectives or actions of carers or bystanders (n=5), all were qualitative. All studies reported that the influence of carers and bystanders was towards the use of ambulances, rather than against.

### Formal carer responsibilities

The actions of those with formal caring responsibilities, including those in loco parentis of children are often recognised in the literature as important components of the decision to seek help from ambulances. Several studies highlight the lower threshold of medical ‘risk’ tolerated by formal care-givers in these situations,^23^ ^24^ ^27^ ^29^ with the default action during illness leaning towards ambulance care. Often, the voices of the relatives can be louder than those of the patient, who may be led down a less appropriate decision-making pathway by well meaning but misinformed relations.^27^ Two thematic studies make reference to the notion that the sick relative is perceived to be less able to make appropriate decisions about their care by virtue of their condition,^27^ ^29^ resulting in de facto vicarious decision-making. Indeed, one interview study illustrates that the roles of relatives and formal carers continue to shape negotiations about non-conveyance even after assessment by the ambulance crew.^29^ Relatives and formal carers appear to be powerful negotiators who often support the course of action that carries least risk, even though this may not be the most clinically appropriate, or even in line with the patient's preferred priorities.

### Bystander actions

Two studies also note that unrelated bystanders often initiate calls for low acuity situations that occur in public places.^23^ ^31^ The initiator of the ambulance call is important in determining how likely it is to be medically necessary, with some relationship between bystander knowledge of basic first aid and the ability to determine what is likely to be a relatively minor illness or injury.^23^ Members of the public appear to be able to appropriately determine the need for an emergency ambulance when they rate their condition as causing ‘severe pain’ or being ‘potentially life-threatening’.^23^ However, they can be influenced by bystanders towards requesting ambulance help if their condition is more minor.^27^

### Population-level factors (n=11 studies)

A number of studies (n=11 papers) directly analyse the impact of socioeconomic status and demographic variations in access to and use of ambulance services for non-serious conditions, using either self-reported questionnaire data or routinely collected data at the time of care episodes. Comparisons are complex, as these studies encompass a variety of global health systems and health economies, with varying levels of correcting for confounding. Some comparative studies use the regional level as the unit of comparison, some use national, and some international perspectives. Broadly, markers traditionally associated with deprivation were associated with increased ambulance use for non-urgent conditions. No UK-based studies exploring socioeconomic variation in detail met the inclusion criteria.

### Demographics

Globally, ambulance usage for non-serious conditions shows considerable variation according to demographic factors. In Japan, the two studies that met the eligibility criteria used self-reported postal questionnaire methodologies to identify patterns of ambulance usage. Men and the elderly are more likely to use emergency ambulances for non-urgent problems.^32^ In addition, those who lived alone or without the family support network of younger relatives were more likely to call for an ambulance when questioned about hypothetical situations that did not require an emergency response.^33^ The authors conclude that access to support from younger family members with positive attitudes to assisting the elderly appears to be associated with seeking treatment from primary care services rather than emergency services.

Similar findings were reported in several US studies, demonstrating old age^16^ ^34^ and male gender^17^ to be associated with increased inappropriate ambulance use. A separate US study approached the issue from a different perspective, exploring willingness to consider alternatives to emergency ambulances for acute care,^35^ by interviewing patients at the time of presentation to emergency services. This study supported the above findings, by showing a willingness to consider that non-ambulance care was associated with adults of working age (18–65 years) but, in contrast with other studies, unemployment.

Minority ethnicity was positively associated with ambulance use in two US studies^17^ ^34^ both of which were retrospective analyses of routinely collected national data sets.

Being located in a rural area showed mixed relationships with ambulance usage. A focus group and patient interview study on urgent care-seeking behaviour in Scotland indicated those in the more rural areas would delay seeking urgent ambulance assistance due to their remoteness, preferring a more ‘wait and see’ approach.^28^ However, a study in the USA that used a substantial national dataset of over 16 million ambulance journeys to emergency departments found that urban location had a corrected OR of 1.46 (1.2 to 1.7) of ambulance use.^34^ This was not the case in a study of children set in South Carolina (USA), reporting a higher odds ratio of 1.247 (1.041 to 1.492) of unnecessary ambulance transport in rural areas.^21^ Other studies have shown that patterns of consulting for perceived paediatric emergencies may be different,^24^ so the significance of this variation would require more detailed exploration.

### Socioeconomic status and deprivation

One Japanese study found that those without their own transport were more likely to call ambulances for medically unnecessary situations.^32^ While increasing household income was associated with reducing rates of ambulance use, the demand for ambulances did not decrease consistently with a theoretical price paid by the user, suggesting the relationship is more complex than monetary cost alone. Socioeconomic factors associated with ambulance use in US-based studies include living in non-private (ie, social) housing,^20^ unemployment^35^ and homelessness^17^—particularly in the elderly, where one retrospective analysis of demographic factors recorded by Fire Department paramedics concluded an eightfold increase in emergency ambulance use compared with matched controls. A self-administered cross-sectional survey of health-seeking behaviour in the USA demonstrated that higher educational level was associated with reduced use of ambulance services,^16^ and poverty (as defined by total household income) was associated with increased use.

### Health insurance status

Several (n=4) US-based studies included health insurance status in their analysis of ambulance usage. For non-urgent conditions, ambulance usage was associated with social healthcare insurance in all three studies. Three studies found that being insured within the Medicaid programme (typically associated with low-income individuals and families) was associated with ambulance use,^16^ ^21^ ^36^ whereas one study found the same for Medicare (typically associated with the elderly and disabled).^34^ As such, all four showed an indirect association with lower socioeconomic and health quality status.

### Healthcare infrastructure factors (n=10)

A mixture of qualitative and quantitative studies evaluated access to and satisfaction with the urgent care health infrastructure, either regionally or nationally.

### Experience, satisfaction and misconceptions of health infrastructure

Five qualitative papers directly explored patient-reported perceptions of the capacity and capability of the health infrastructure to deal with their perceived or actual problems.^24^ ^27^ ^31^ ^37^ ^38^ Several of these reported themes of misconception about the level or type of care that could be delivered by the ambulance crews^24^ ^27^ or receiving emergency units.^31^ ^37^ One study identified reassurance as a key element of the ambulance response that patients valued,^38^ which links with another study that identified the amount of time spent and thoroughness of the clinical assessment as features that differentiate an ambulance response from an alternative urgent care avenue.^24^ Satisfaction scores reduced sequentially with the number of different services a patient contacted prior to definitive care.^37^

### Presence of a primary care model

The presence (or absence) of a primary care model of health within the wider infrastructure was identified in varying degrees as related to ambulance use. One Swedish study estimates that urgent primary care services handle 42 500 potentially life-threatening presentations annually.^39^ In the Netherlands, a cross-sectional study concluded that 88% of all out-of-hours urgent care requests were handled by primary care. Japanese data indicates that the relatively limited existence of primary care and social isolation are important themes in driving inappropriate and expensive ambulance use.^33^ Those with access to urgent primary care services in Japan were less likely to use an ambulance inappropriately in hypothetical scenarios.^32^

### Role of other services in unmet needs

Several UK studies explored, qualitatively and quantitatively, patients’ use of services prior to contact with the ambulance service. Ambulances called after a triage-contact with NHS 24 (a telephone triage service) were more likely to be classified as inappropriate.^40^ One paper reports that 68% of service users had contact with more than one service during their urgent care need, with an average of two contacts per episode.^37^ Several studies specifically explored patient actions in the lead-up to an emergency call. In general, where primary care services existed, the majority of patients who ultimately were categorised as inappropriately receiving ambulance care had attempted to contact their GP beforehand.^24^ ^37^ ^41^ One study found that 1 in 20 calls required either ‘general assistance’, or were not regarding any illness or injury at all.^42^ Social care was usually the unmet need.

### Challenges faced by health professionals (n=21)

Of the included evidence, 21 papers referred primarily or substantially to health professionals’ perspectives in the assessment of need for an ambulance.

### Inappropriateness as a concept

The assessment of ‘inappropriateness’ of an ambulance contact is complex and varied in the included evidence; a result of methodological limitations and conceptual variation. The majority of studies sought to determine ‘inappropriateness’ retrospectively from case notes, using semiobjective scoring or coding systems. Assessments were performed by emergency department clinicians^20^ ^21^ ^23^ ^26^ ^27^ ^40^ ^43^ and pre-hospital staff.^17–19^ ^21^ ^26^ ^27^ ^29^ ^33^ Two included assessments of severity of illness undertaken by primary care staff.^39^ ^41^ The professional background and seniority of staff varied. One meta-analysis of US paramedic decision-making reports an aggregate negative predictive value of 0.912 (0.707 to 0.978) for ambulance paramedic determination of necessity for transport at the scene, when compared with hospital physician assessment.^44^

Only one US study involving children utilised medical necessity criteria agreed at a consensus conference to make these assessments.^21^ Other studies used a mixture of one or more professional opinions,^18^ ^19^ ^23^ ^26–28^ coding systems, or scores based on physiological parameters or clinical conditions,^17^ ^20^ ^39^ standardised diagnosis codes such as the International Classification of Primary Care,^41^ or custom designed instruments or topic guides to explore provider perceptions of necessity.^29^ ^33^

Two comprehensive reviews explored the concept of ‘inappropriateness’ specifically and qualitatively.^5^ ^22^ One reported the theme of inappropriateness as divided into two cohorts: those not experiencing a health emergency, and those experiencing an emergency but who do not seek ambulance care when they should.^22^ The other review concludes that assessment of appropriateness based on information available after clinical assessment will overestimate ‘inappropriate’ use, and neglects the complex psychosocial context of the request for help.^5^

### Fitness for purpose of the triage process

Several studies directly or indirectly address the role of telephone triage systems in supporting or confounding both patient and clinical decision-making. A UK study reports that 26% of ambulance calls coded as the highest clinical priority at triage resulted in no patient being conveyed to hospital.^45^ Another study highlighted the need for good real-time links between ambulance and primary care triage systems, due to the cross-over of calls.^39^ Other qualitative studies report the theme of validation of the urgent need for care which can be inferred from triage.^24^ ^25^

## Discussion

This review has highlighted some important factors that may impact ambulance use for primary care problems in a variety of international settings. Studies, in general, support an association with certain sociodemographic factors including minority ethnic category, lower income, public insurance (where this exists) and increasing age. Broadly, the individual patients’ social circumstances (including the structure of the household), their perceptions of urgency and the shaping influence of care-givers and bystanders appear to have more impact on excess ambulance use than the actual clinical problem or final diagnosis.

This analysis of the literature reveals a complex (and often unclear) relationship between ‘urgency’ of the clinical problem and ‘appropriateness’ of the use of ambulances. Primary care sensitive problems are not consistently defined in the literature, and are often categorised interchangeably with ‘non-emergency’ or ‘non-serious’ problems. Indeed, whether a problem is ‘primary care sensitive’ or not appears to vary depending on how accessible and developed a primary care model is, and on the capacity to respond to demands made of it. Developing a uniform definition of a ‘primary care sensitive problem’ applicable across contrasting ambulance systems presents a challenge for further research.

Mapping the literature reveals that many of the conflicts that exist around whether ambulance use was ‘appropriate’ or otherwise originate from contrasts between prospective and retrospective determinations. Those studies that seek to assign a measure of ‘appropriateness’ usually do so after clinical treatment and with the benefit of a clinical diagnosis, and almost universally from the healthcare provider's perspective. The heterogeneity of ambulance systems in operation worldwide means ‘appropriateness’ can be evaluated from a variety of social and clinical perspectives with different conclusions. The presence of an established primary care model with dedicated, accessible urgent care channels does appear to have a positive impact. However, the difficulty in determining ‘appropriateness’ highlights the compounding effect of a concept that is situation sensitive and varies across international contexts, and the methodological limitations of studies that blur the notions of ‘unnecessary’ and ‘primary care sensitive’. Future work needs to focus on defining these overlapping but conceptually distinct entities.

The role of triage also appears complex. The literature suggests that—where detailed primary care-focussed triage exists—it can identify, redirect or often completely manage ‘primary care sensitive’ contacts. However, the literature also indicates that triage must be sensitive to patient and care-giver perceptions of risk, which can be magnified when presented with unforeseen and non-specific health symptoms. Recurring messages in the literature are patient and carer uncertainty around urgency, the fear of harm if treatment is delayed and the value placed on clinical assessment for reassurance. Bystander and care-giver decisions appear particularly moderated by perceptions of risk. While some studies suggest that public education can be helpful in reducing ambulance usage, it appears unlikely that education alone will substantially reduce this perception. Strategies that help patients and care-givers to mitigate perceived risk are likely to be beneficial.

This is the first comprehensive mapping review exploring why primary care sensitive problems present to ambulance services specifically. This is a highly inclusive systematic review conducted in accordance with a prospectively published protocol, encompassing a broad range of qualitative, quantitative and mixed methods studies and reviews with an interpretive element. This heterogeneity necessarily requires a narrative synthesis and prevents meta-analysis, and may limit the applicability of some results to contrasting ambulance systems. Studies vary in reporting quality, and although no studies were excluded on the grounds of quality alone, variation in study design and reporting meant no statistical analysis of quantitative data was possible. While there are obvious limitations in attempting to apply a standardised quality measure across such a variety of study types, the use of established tools enabled a consensus discussion within the research team of what a ‘fundamentally flawed’ study would look like, and was felt to be an appropriate compromise between robustness and pragmatism. Additionally, while it is known that children account for a substantial proportion of the urgent care workload, the majority of included studies were conducted on adult populations, limiting the conclusions for the paediatric subset.

Future work needs to focus on more precisely defining ‘primary care sensitive’ problems within the specific context of ambulance services, and on understanding how to respond to the complex psychosocial perception of urgency that appears to be driving increasing ambulance use. Exploring strategies to assist patients and bystanders in mitigating their perceptions of risk, and how these can be achieved through the triage process, is also likely to be important. Research and policy needs to acknowledge the frustrations felt by healthcare providers about so-called ‘unnecessary’ ambulance use, but be sensitive to the idea that patients and carers often do not know exactly what type of help they need when they contact urgent care services.
